# TWEAK activation of the non-canonical NF-κB signaling pathway differentially regulates melanoma and prostate cancer cell invasion

**DOI:** 10.18632/oncotarget.13034

**Published:** 2016-11-03

**Authors:** Cheryl L. Armstrong, Rebeca Galisteo, Sharron A.N. Brown, Jeffrey A. Winkles

**Affiliations:** ^1^ Department of Surgery, University of Maryland School of Medicine, Baltimore, MD, USA; ^2^ Center for Vascular and Inflammatory Diseases, University of Maryland School of Medicine, Baltimore, MD, USA; ^3^ Marlene and Stewart Greenebaum Comprehensive Cancer Center, University of Maryland School of Medicine, Baltimore, MD, USA

**Keywords:** TWEAK, Fn14, invasion, NF-κB, melanoma

## Abstract

Tumor necrosis factor-like weak inducer of apoptosis (TWEAK) is a multifunctional cytokine that binds with high affinity to a plasma membrane-anchored receptor named Fn14. Both TWEAK and Fn14 expression has been detected in human cancer tissue, and studies have shown that TWEAK/Fn14 signaling can promote either “pro-cancer” or “anti-cancer” cellular effects *in vitro*, depending on the cancer cell line under investigation. In this study, we engineered murine B16 melanoma cells to secrete high levels of soluble TWEAK and examined their properties. TWEAK production by B16 cells preferentially activated the non-canonical NF-κB signaling pathway and increased the expression of several previously described TWEAK-inducible genes, including Fn14. TWEAK overexpression in B16 cells inhibited both cell growth and invasion *in vitro.* The TWEAK-mediated reduction in B16 cell invasive capacity was dependent on activation of the non-canonical NF-κB signaling pathway. Finally, we found that this same signaling pathway was also important for TWEAK-stimulated human DU145 prostate cancer cell invasion. Therefore, even though TWEAK:Fn14 binding activates non-canonical NF-κB signaling in both melanoma and prostate cancer cells, this shared cellular response can trigger a very different downstream outcome (inhibition or stimulation of cell invasiveness, respectively).

## INTRODUCTION

Tumor necrosis factor (TNF)-like weak inducer of apoptosis (TWEAK), a member of the TNF superfamily of multifunctional cytokines, acts on cells via binding to a small cell surface receptor named fibroblast growth factor-inducible 14 (Fn14) [1, 2]. TWEAK is initially synthesized as a type II transmembrane protein, but it can undergo intracellular furin cleavage, which releases a soluble cytokine that can act on cells in an autocrine or paracrine manner [3, 4]. Membrane or soluble TWEAK binding to Fn14 promotes receptor trimerization, TNF receptor associated factor (TRAF) association, and activation of various downstream signaling pathways, including the canonical (classical) and non-canonical (alternative) NF-κB pathways [5–11]. Briefly, in the canonical pathway, which is activated by many extracellular stimuli (e.g., inflammatory cytokines, bacterial lipopolysaccharide (LPS), UV radiation), the IKK complex protein IKKβ phosphorylates IκBα, which triggers IκBα polyubiquitination and degradation, thereby enabling NF-κB dimers (e.g., RelA (p65)/p50) to translocate to the nucleus and regulate gene expression. In the non-canonical pathway, which is primarily activated by certain TNF family members, the kinase NIK phosphorylates IKKα, which in turn phosphorylates NF-κB2 (also known as p100), leading to p100 ubiquitination and partial degradation by the proteasome to generate p52. RelB/p52 heterodimers then translocate to the nucleus and regulate gene expression [12–14].

TWEAK:Fn14 engagement *in vivo* is thought to play an important role in tissue repair and regeneration following acute injury, and numerous studies have indicated that sustained Fn14 activation can promote the pathological tissue remodeling associated with chronic inflammatory, autoimmune, and neurodegenerative diseases [1, 2, 15]. Accordingly, a number of TWEAK-targeted therapeutic agents are in pre-clinical or clinical development for these conditions [2, 16].

TWEAK/Fn14 axis signaling has also been implicated in cancer, the second leading cause of death in the USA [17]. While TWEAK and Fn14 gene expression is low in normal healthy tissues, increased expression of one or both of these genes has been detected in many solid primary tumor types and tumor metastases [1, 18–20]. For example, TWEAK is highly expressed in kidney [21, 22], liver [23], colon [21, 24, 25], ovarian [26], esophageal [27], and pancreatic [27] cancer. TWEAK is a pro-angiogenic [21, 28, 29] and pro-inflammatory [30–33] factor *in vivo*, so it could promote tumor vascularization and inflammation. Additionally, studies have shown that TWEAK-triggered Fn14 activation in cancer cells themselves can stimulate either “pro-tumorigenic/metastatic” or “anti-tumorigenic/metastatic” cellular responses *in vitro*, depending on the cell line under investigation. In regard to the “pro-tumorigenic/metastatic” responses, TWEAK has been shown to induce cancer cell growth [23], migration [34–38], invasion [11, 35, 39], and chemotherapy drug resistance [40–43]. In consideration of these findings, an anti-TWEAK neutralizing monoclonal antibody (mAb) named RG7212 was developed for potential use in cancer patients. This agent inhibited tumor growth in preclinical xenograft models [44] and in a phase I clinical trial it was able to induce prolonged stable disease in 15 patients (28%) with one malignant melanoma patient showing evidence of tumor regression [19]. However, this therapeutic strategy, as well as others designed to inhibit the TWEAK:Fn14 interaction [27, 45, 46], may need to be re-considered since, as mentioned above, TWEAK:Fn14 engagement in certain cancer cell lines can induce beneficial “anti-tumorigenic/metastatic” cellular responses. For example, TWEAK is a pro-apoptotic factor for some cancer cell lines [3, 24, 47, 48] and TWEAK treatment of HCT116 colorectal cancer cells inhibits invasive capacity [25]. Accordingly, several groups have developed agonistic Fn14 mAbs for cancer therapy [18, 49–53] and one antibody, named PDL192, has been evaluated for safety in a phase I clinical trial [54]. In summary, although the TWEAK/Fn14 signaling axis appears to be a promising therapeutic target for multiple cancer types, more studies are warranted in order to understand the full spectrum of TWEAK-mediated pro- and anti-cancer cell effects.

In the current study, we engineered murine B16 melanoma cell lines that overexpressed the soluble TWEAK (sTWEAK) isoform and compared their biological properties to matched control cell lines with low levels of endogenous TWEAK expression. The TWEAK-overexpressing cells had elevated levels of Fn14 expression and higher amounts of several chemokines were detected in conditioned media. Also, TWEAK overexpression in B16 cells (i) inhibited cell growth *in vitro* but not *in vivo,* (ii) had no significant effect on cell migration, (iii) significantly reduced cell invasion. Furthermore, this latter effect depended, at least in part, on activation of the non-canonical NF-κB signaling pathway. Finally, in studies using human DU145 prostate cancer cells, we found that non-canonical NF-κB signaling pathway activation was also important for TWEAK-stimulated cell invasion. These findings demonstrate that TWEAK/Fn14 axis-triggered non-canonical NF-κB signaling pathway activation in cancer cells can positively or negatively regulate cellular invasive activity, depending on the particular cancer cell line under investigation.

## RESULTS

### Constitutive sTWEAK overexpression in murine B16 melanoma cells increases Fn14 and chemokine expression

We chose to study the effects of human sTWEAK overexpression in melanoma cells in consideration of data indicating that TWEAK/Fn14 pathway activation may play a role in human metastatic melanoma [19, 44, 55]. However, since most human melanoma cells in culture express high levels of Fn14 [55], which could initiate TWEAK-independent Fn14 signaling [56], we selected murine B16-BL6 melanoma cells for our experiments. These cells express low basal levels of both TWEAK and Fn14 [44]. Also, B16 cells are syngeneic with C57BL/6 mice [57, 58], so their growth following subcutaneous implantation can be evaluated in an immunocompetent host. Finally, murine cells can be used to study the effects of human TWEAK overexpression since our group and others have demonstrated that human TWEAK can bind with high affinity to the murine Fn14 protein [59–61].

**Figure 1:**
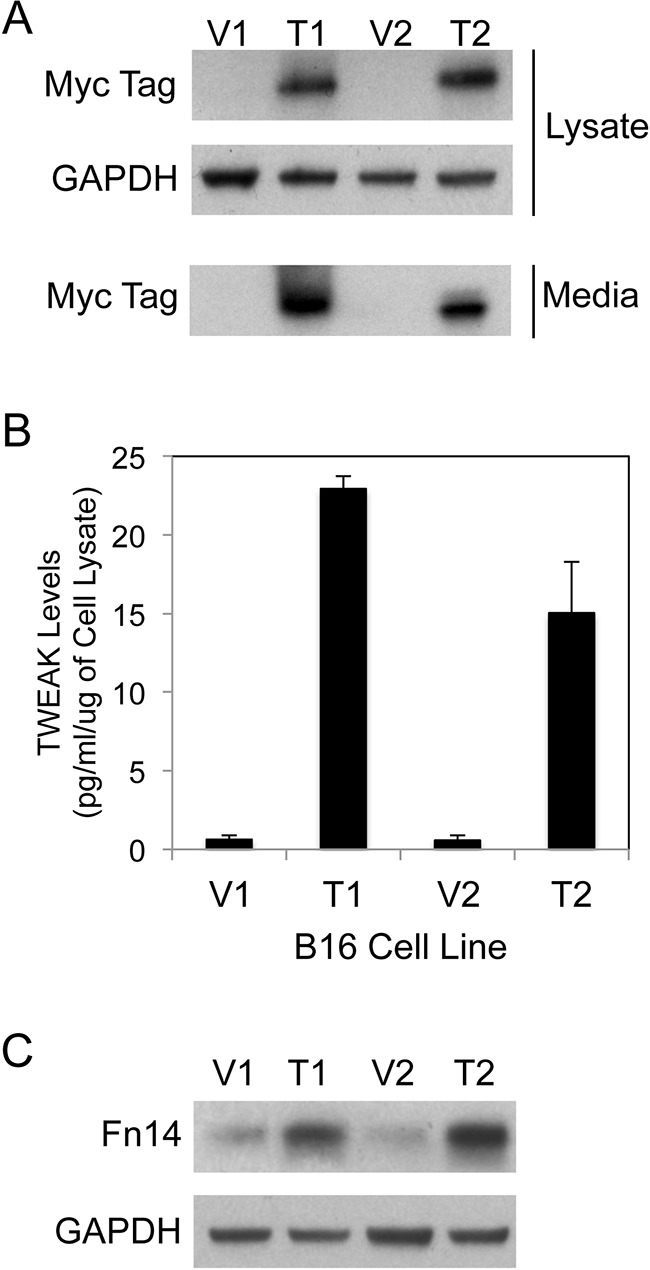
Human sTWEAK overexpression in murine B16 melanoma cells increases Fn14 expression **A.** Vector control (V1, V2) and TWEAK-myc plasmid-transfected (T1, T2) B16 clonal cell lines were harvested and cell lysate and conditioned media samples were prepared. TWEAK and GAPDH levels were analyzed by Western blotting using anti-myc tag and anti-GAPDH antibodies, respectively. This Western blot was done three times. **B.** Human sTWEAK levels in conditioned media samples collected from the four B16 cell lines were determined by ELISA. The results shown are the combination of 2 independent experiments for V1/T1 and 3 independent experiments for V2/T2. **C.** The four B16 cell lines were harvested and Fn14 and GAPDH levels were evaluated by Western blot analysis. This Western blot was done three times.

Parental B16-BL6 cells were transfected with two different mammalian expression vectors (pSecTag, pcDNA6) and their corresponding TWEAK expression constructs and individual clonal cell lines were isolated by drug selection. The sTWEAK protein contained an N-terminal myc tag in order to facilitate its detection in cells and conditioned media by Western blot analysis. One pair of vector (V)-transfected and TWEAK (T)-overexpressing clonal cell lines were selected from each expression construct type for further characterization (denoted V1, T1 and V2, T2). TWEAK expression and secretion by the T1 and T2 cell lines was confirmed by Western blot analysis using cell lysates and conditioned media samples, respectively (Figure 1A). Also, the amount of TWEAK in conditioned media collected from the four cell lines was determined using a solid-phase, sandwich ELISA that only detects human TWEAK. We found that the T1 and T2 culture media samples contained high levels of sTWEAK (Figure 1B).

As mentioned above, it has been reported that parental B16-BL6 cells express low levels of Fn14 [44]. Nevertheless, we postulated that sTWEAK secreted from the T1 and T2 cell lines might bind whatever Fn14 was on the surface of these cells, activate signaling pathways, and ultimately change the cellular gene expression profile. Since TWEAK treatment of glioma [62], prostate cancer [48] and melanoma [55] cells *in vitro* has been shown previously to increase Fn14 gene expression, we first analyzed Fn14 protein levels in the four B16 cell lines using Western blot analysis. Fn14 expression was elevated in the TWEAK-overexpressing cell lines compared to their corresponding empty-vector control cell lines (Figure 1C). To confirm that TWEAK overexpression in B16 cells was indeed having an impact on gene expression, we examined the expression level of 111 different murine cytokines, chemokines and growth factors using B16 cell conditioned media samples and an antibody array. We detected increased levels of CCL2/JE/MCP-1, CCL5/RANTES, and CXCL1/KC protein in the TWEAK-overexpressing T1 and T2 conditioned media samples compared to their respective V1 and V2 control cell conditioned media samples (Supplementary Figure S1). Taken together, these results indicate that TWEAK overexpression in B16 cells results in TWEAK:Fn14 engagement and downstream changes in gene expression.

### TWEAK overexpression in B16 cells inhibits cell growth *in vitro* but not *in vivo*

**Figure 2:**
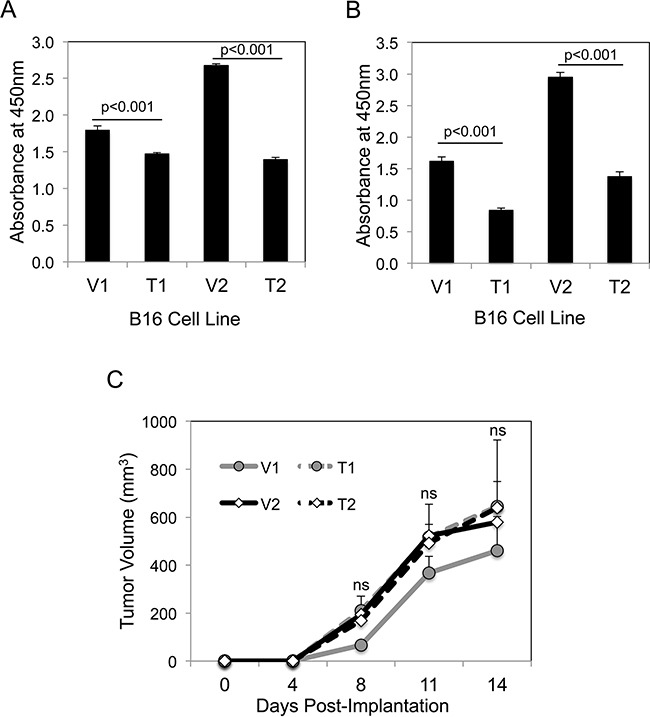
TWEAK overexpression in B16 cells reduces growth *in vitro* but not *in vivo* **A.** The four B16 cell lines were cultured for 72 hr in low serum media (0.5% FBS) and cell growth was assayed using WST-1 reagent. The values shown are mean ± SEM of four replicates per cell line. Significance was measured by Student's t-test. This growth assay was performed four times and the results of one representative experiment are shown here. **B.** The four B16 cell lines were cultured for 72 hr in normal growth media (10% FBS) and cell growth was assayed as above. The values shown are mean ± SEM of four replicates per cell line. Significance was measured by Student's t-test. This growth assay was performed four times and the results of one representative experiment are shown here. **C.** The four B16 cell lines were injected subcutaneously into syngeneic C57BL/6 mice (n = 7 animals per cell line) and tumor growth was monitored over time. The values shown are the mean ± SEM. Significance was measured by Student's t-test (ns = not significant; p > 0.05). This *in vivo* experiment was conducted twice and the results of one representative experiment are shown here.

We first investigated whether sTWEAK overexpression altered B16 cell growth *in vitro* using the WST-1 cell proliferation assay. The TWEAK-overexpressing cell lines had decreased proliferative potential compared to their corresponding empty-vector cell lines in both low serum (Figure 2A) and high serum (Figure 2B) culture conditions. The parental B16 cell line and the vector cell lines had similar growth properties; in addition, TWEAK treatment of parental B16 cells had no significant effect on cell growth (data not shown). We then determined if a similar difference in growth potential could be detected when the four B16 cell lines were implanted subcutaneously in syngeneic C57BL/6 mice. We found that TWEAK overexpression did not significantly alter B16 cell growth *in vivo* (Figure 2C). The parental B16 cell line had similar growth properties as the engineered B16 cell lines *in vivo* (data not shown).

### TWEAK overexpression in B16 cells has no effect on cell migration

**Figure 3:**
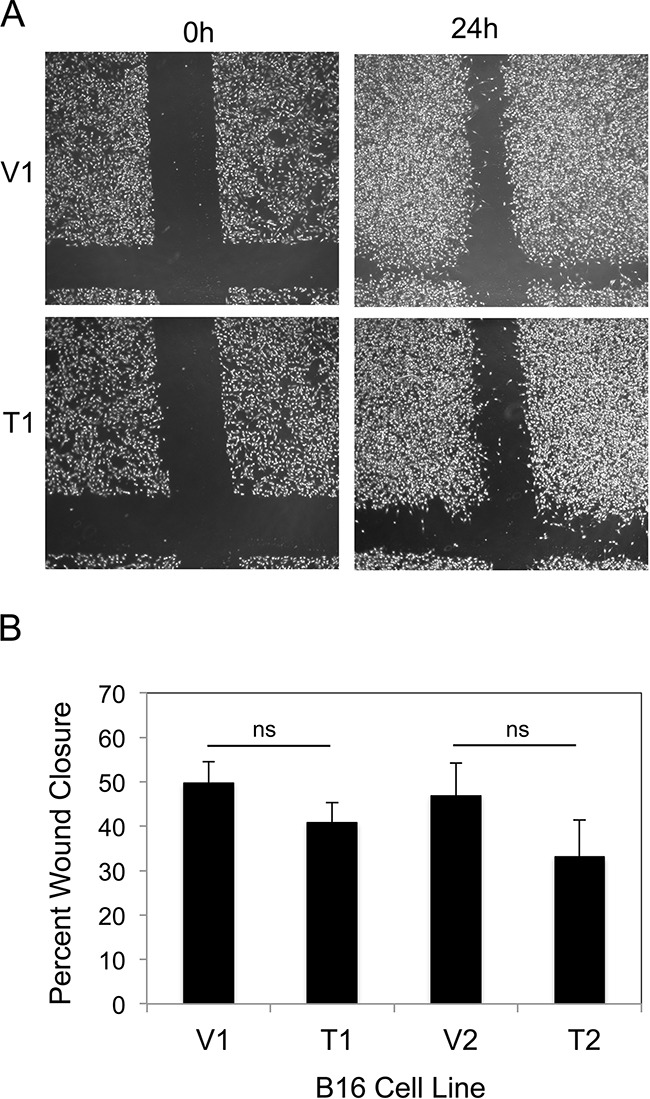
TWEAK overexpression does not alter B16 cell migration **A.** The four B16 cell lines were grown to confluence and a scratch wound was made using a pipette tip. Wound closure was monitored by light microscopy and representative photographs (x10 magnification) of the V1 and T1 cell lines at 0 and 24 hr after wounding are shown. **B.** Wound width was calculated at 0 and 24 hr for all four B16 cell lines and the difference plotted as percentage wound closure. The values shown are the mean ± SEM for three replicates per cell line. Significance was measured by Student's t-test (ns = not significant; p > 0.05). The migration assay was performed three times and the results of one representative experiment are shown here.

We compared the migratory potential of the four B16 cell lines using the scratch wound assay. Confluent cell monolayers were scratched in the shape of a cross using a pipette tip and photographs were taken prior to and 24 hr after wounding (Figure 3A). Wound width was measured and percentage wound closure was calculated. We found that TWEAK overexpression did not significantly alter B16 cell migration using this assay (Figure 3B).

### Both sTWEAK overexpression and recombinant TWEAK treatment inhibit B16 cell invasion

**Figure 4:**
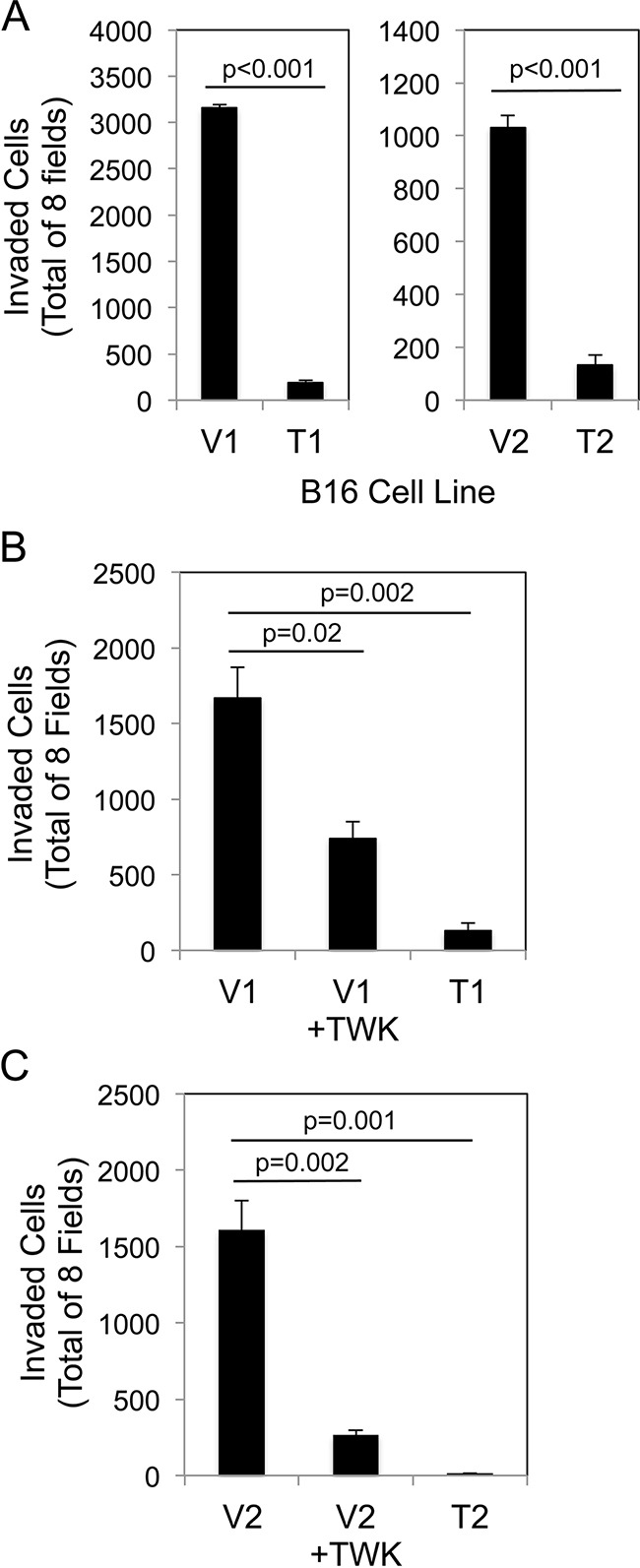
Both TWEAK overexpression in B16 cells and exogenous TWEAK treatment of B16 cells inhibits cell invasion **A.** The four B16 cell lines were evaluated for invasive capacity using the Matrigel invasion assay. The number of invaded cells for each invasion chamber was determined from photographs of eight randomly chosen fields. The values shown are the mean ± SEM for three chambers per cell line. Significance was measured by Student's t-test. **B.** V1 cells were either left untreated or treated with 200 ng/ml of TWEAK (TWK) for 24 hr prior to plating in Matrigel invasion chambers. T1 cell invasion was also evaluated. The values shown are the mean ± SEM for three chambers per condition. Significance was measured by Student's t-test. **C.** V2 cells were either left untreated or treated with 200 ng/ml of TWEAK (TWK) for 24 hr prior to plating in Matrigel invasion chambers. T2 cell invasion was also evaluated. The values shown are the mean ± SEM for three chambers per condition. Significance was measured by Student's t-test. All invasion assays were performed at least two times and the results of one representative experiment are shown here.

We investigated whether sTWEAK overexpression in B16 cells altered basal invasive activity using modified Boyden chambers coated with Matrigel, a solubilized basement membrane preparation extracted from the EHS mouse sarcoma [63]. Both of the TWEAK-overexpressing B16 cell lines exhibited significantly decreased invasive potential compared to their corresponding empty-vector B16 cell lines (Figure 4A). To determine if this reduced cell invasion capacity was a direct response to TWEAK:Fn14 engagement, the two empty-vector cell lines were treated with recombinant human TWEAK for 24 hr and then Matrigel invasion assays were conducted. TWEAK treatment of the V1 (Figure 4B) and V2 (Figure 4C) cell lines reduced invasion by ~56% and ~84%, respectively, compared to untreated cells.

### Constitutive sTWEAK overexpression in B16 cells promotes non-canonical NF-κB pathway activation, and this effect can be inhibited by Fn14 siRNA transfection

**Figure 5:**
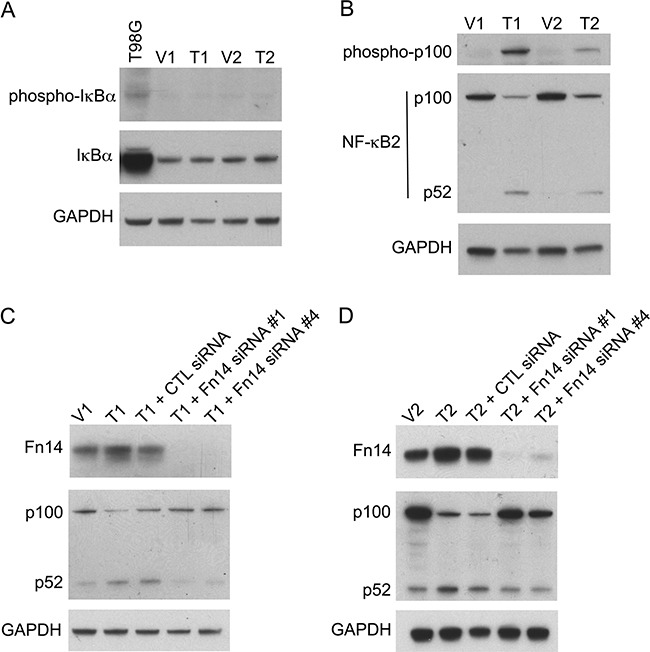
TWEAK overexpression in B16 cells activates the non-canonical NF-κB pathway and Fn14 depletion attenuates this effect **A.** Human T98G cells (positive control) and the four engineered B16 cell lines were harvested and phospho-IκBα, IκBα, and GAPDH levels were evaluated by Western blot analysis. This Western blot was done twice. **B.** The four B16 cell lines were harvested and phospho-p100, NF-κB2 (p100/p52) and GAPDH levels were evaluated by Western blot analysis. This Western blot was done three times. **C.** V1 and T1 cells were either left untreated or T1 cells were transiently transfected with control (CTL) siRNA, Fn14 siRNA #1 or Fn14 siRNA #4 for 48 hr. Cells were harvested and Fn14, NF-κB2 (p100/p52) and GAPDH levels were evaluated by Western blot analysis. This Western blot was done once. **D.** V2 and T2 cells were either left untreated or T2 cells were transiently transfected with control (CTL) siRNA, Fn14 siRNA #1 or Fn14 siRNA #4 for 48 hr. Cells were harvested and Fn14, NF-κB2 (p100/p52) and GAPDH levels were evaluated by Western blot analysis. This Western blot was done once.

TWEAK:Fn14 engagement can activate several intracellular signal transduction pathways, including the Ras/Raf/MEK/ERK pathway [21, 64], the PI3K/Akt pathway [41], and both the canonical and non-canonical NF-κB pathways [5–11]. In order to investigate if one or more of these signaling pathways were critical for TWEAK inhibition of B16 cell invasion we first determined whether any of them were constitutively activated in the TWEAK-overexpressing B16 cell lines. Western blot analysis was conducted using antibodies that are commonly used to monitor the cellular status of the four signal transduction cascades listed above. We found that neither ERK1/2 nor Akt was preferentially phosphorylated (activated) in the TWEAK-overexpressing cell lines compared to the empty-vector cell lines, indicating that these two pathways were unlikely to play a role in TWEAK regulation of B16 cell invasiveness (data not shown). Also, TWEAK overexpression did not increase IκBα phosphorylation (Figure 5A), reduce IκBα levels (Figure 5A), nor increase p65 phosphorylation (data not shown), three molecular changes associated with canonical NF-κB pathway activation. However, the TWEAK-overexpressing cell lines did exhibit constitutive non-canonical NF-κB pathway activation, as monitored by assaying the phosphorylation status of NF-κB2 (p100) and the proteolytic processing of p100 to p52 (Figure 5B). To confirm that TWEAK:Fn14 engagement in the T1 and T2 cell lines was triggering non-canonical NF-κB signaling, we depleted Fn14 levels in these cells using two different siRNAs and monitored p100 processing by Western blot analysis. We found that Fn14-depleted cells had reduced non-canonical pathway activation in comparison to both untransfected or control siRNA-transfected cells (Figure 5C, D).

### TWEAK treatment of B16 cells also promotes non-canonical NF-κB pathway activation

To test if exogenously added TWEAK also preferentially activated the non-canonical NF-κB pathway in B16 cells, the empty-vector V1 cell line was treated with recombinant TWEAK for various lengths of time, cells were harvested, and both canonical and non-canonical NF-κB pathway activation was evaluated by Western blot analysis. TWEAK treatment did not reduce IκBα levels, which occurs during canonical pathway activation (Supplementary Figure S2A). However, we detected increased p100 phosphorylation and increased p100 to p52 processing at 2 hr and 4 hr post-TWEAK addition, respectively, demonstrating that TWEAK exposure was stimulating non-canonical NF-κB pathway activation (Supplementary Figure S2B).

### TWEAK overexpression-triggered non-canonical NF-κB signaling in B16 cells negatively regulates cell invasion

**Figure 6:**
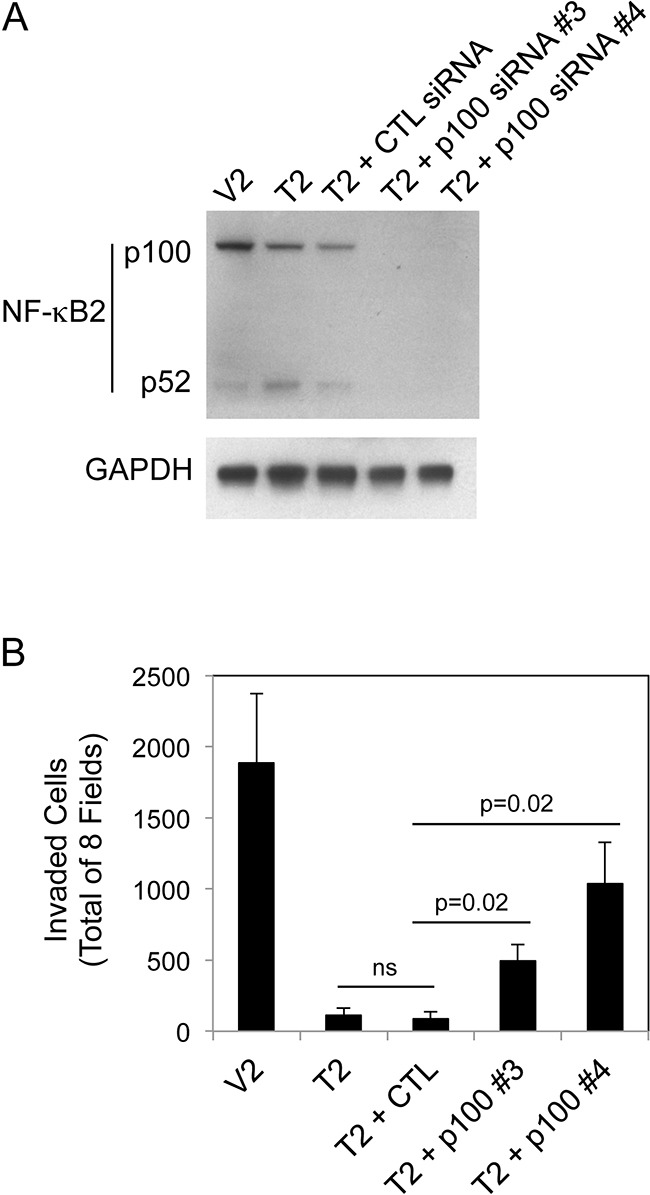
NF-κB2 p100 depletion in B16 T2 cells increases cell invasion **A.** V2 and T2 cells were either left untreated or T2 cells were transiently transfected with control (CTL) siRNA, p100 siRNA #3 or p100 siRNA #4 for 48 hr. Cells were harvested and NF-κB2 (p100/p52) and GAPDH levels were evaluated by Western blot analysis. This Western blot was done twice. **B.** V2 and T2 cells were either left untreated or T2 cells were transiently transfected with control (CTL) siRNA, p100 siRNA #3 or p100 siRNA #4 for 24 hr prior to cell harvest and plating in Matrigel invasion chambers. The number of invaded cells for each invasion chamber was determined from photographs of eight randomly chosen fields. The values shown are the mean ± SEM for three chambers per condition, with each chamber done independently. Significance was measured by Student's t-test (ns = not significant; p > 0.05).

We have shown above that (i) constitutive sTWEAK overexpression in B16 cells reduces invasive capacity, and (ii) TWEAK:Fn14 engagement in B16 cells preferentially activates the non-canonical NF-κB signaling pathway. In consideration of these two findings, we determined if non-canonical NF-κB signaling in TWEAK-overexpressing cells negatively regulated cell invasion. For this analysis, T2 cells were either left untransfected, transfected with control siRNA, or transfected with two different p100-specific siRNAs. Cells were harvested and an aliquot was lysed and used to confirm effective p100 depletion by Western blot analysis (Figure 6A). The remaining cells were used for Matrigel invasion assays. We found that control siRNA transfection did not significantly alter cell invasiveness (Figure 6B). In contrast, p100 depletion in T2 cells using siRNA #3 or #4 increased invasive capacity by ~4.5-fold and ~9.3-fold, respectively, as compared to the untransfected cells. This result indicates that the TWEAK-mediated decrease in B16 cell invasive capacity is due, at least in part, to non-canonical NF-κB pathway signaling.

### TWEAK treatment of human A375 melanoma cells inhibits invasion and activates the canonical and non-canonical NF-κB signaling pathways

**Figure 7:**
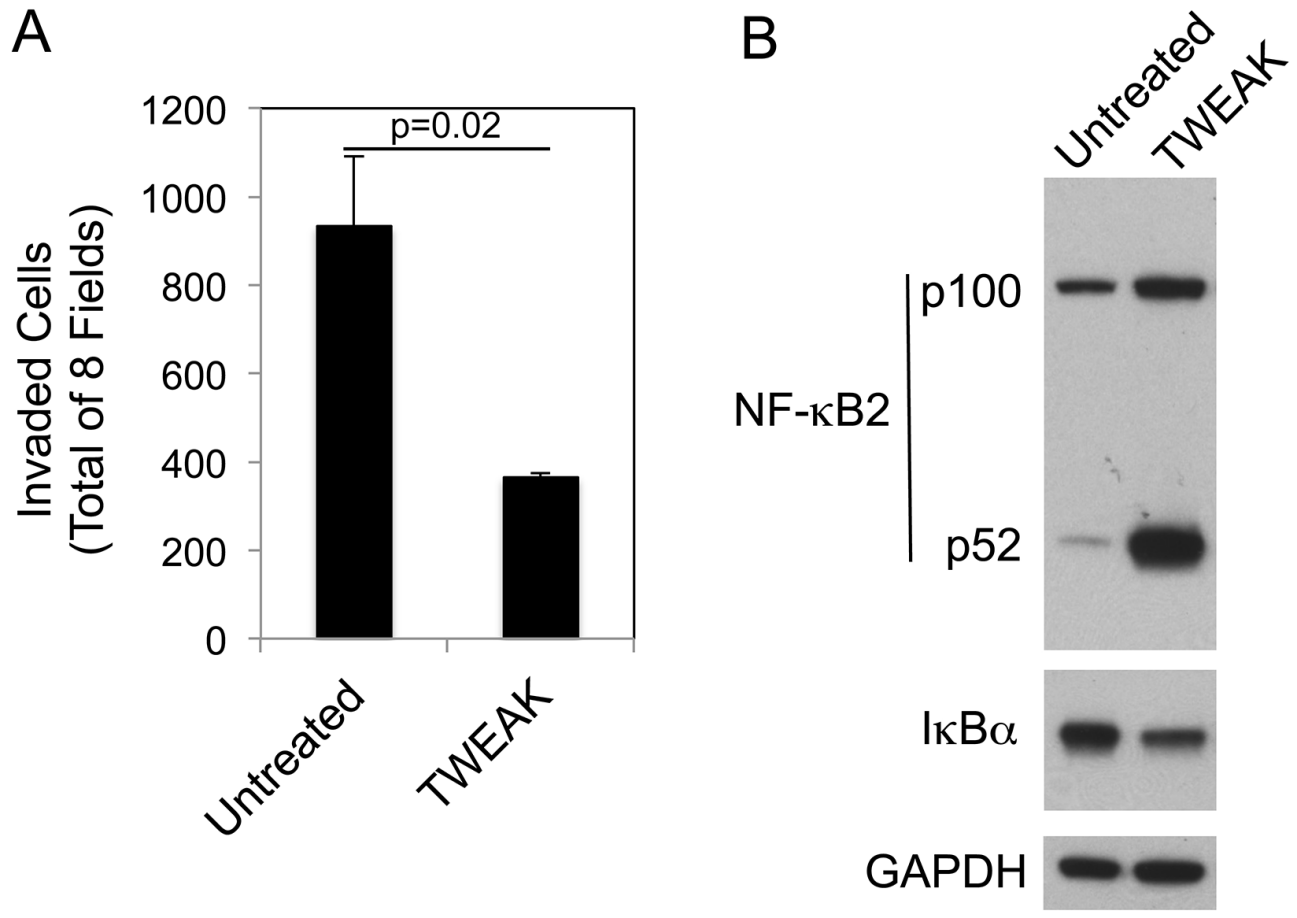
TWEAK treatment of human A375 melanoma cells decreases invasion and activates both the canonical and non-canonical NF-κB pathways **A.** A375 cells were either left untreated or treated with 200 ng/ml of TWEAK for 24 hr prior to harvesting and plating in Matrigel invasion chambers. The number of invaded cells for each invasion chamber was determined from photographs of eight randomly chosen fields. The values shown are the mean ± SEM for three chambers per condition. Significance was measured by Student's t-test. The invasion assay was done twice and the results of one representative experiment are shown here. **B.** A375 cells were either left untreated or treated with TWEAK for 24 hr. Cells were harvested and NF-κB2 (p100/52), IκBα and GAPDH levels were evaluated by Western blot analysis. This Western blot was done twice.

We next determined if the TWEAK-mediated decrease in invasion observed in B16 cells was a unique response restricted to this mouse melanoma cell line or also occurred in human A375 melanoma cells, which express Fn14 [55]. These cells were treated with recombinant human TWEAK for 24 hr and then Matrigel invasion assays were conducted. TWEAK stimulation of A375 cells reduced invasion by ~61% compared to untreated cells (Figure 7A). We then investigated if TWEAK treatment of A375 cells preferentially activated the non-canonical NF-κB pathway, as we observed in the B16 cells. A375 cells were treated with recombinant TWEAK for 24 hr and assayed for canonical and non-canonical NF-κB pathway activation by Western blot analysis. TWEAK treatment increased the NF-κB2 expression level, stimulated p100 to p52 processing, and reduced IκBα levels (Figure 7B), indicating activation of both NF-κB signaling pathways. These results indicate that although TWEAK has different effects on NF-κB signaling when added to mouse or human melanoma cells, it triggers the same downstream outcome - a decrease in invasive capacity.

### TWEAK treatment of human PC-3 and DU145 prostate cancer cells promotes invasion and activates the non-canonical NF-κB pathway

**Figure 8:**
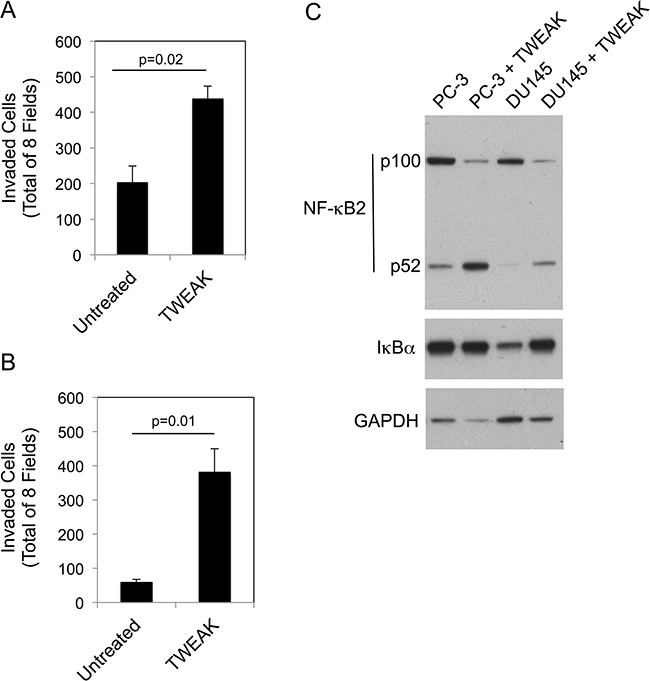
TWEAK treatment of human PC-3 and DU145 prostate cancer cells increases invasion and activates the non-canonical NF-κB pathway PC-3 **A.** and DU145 **B.** cells were either left untreated or treated with 200 ng/ml of TWEAK for 24 hr prior to harvesting and plating in Matrigel invasion chambers. The number of invaded cells for each invasion chamber was determined from photographs of eight randomly chosen fields. The values shown are the mean ± SEM for three chambers per condition. Significance was measured by Student's t-test. The invasion assay was repeated one more time using one chamber per condition with similar results. **C.** PC-3 and DU145 cells were either left untreated or treated with TWEAK for 24 hr. Cells were harvested and NF-κB2 (p100/52), IκBα and GAPDH levels were evaluated by Western blot analysis. This Western blot was done twice.

Although we have shown here that TWEAK:Fn14 engagement in murine or human melanoma cells reduces invasive capacity, other studies have reported that TWEAK exhibits pro-invasive activity when added to certain human cancer cell lines; specifically, BT116 glioblastoma cells [11], HO-8910PM ovarian cancer cells [35] and both PC-3 and DU145 prostate cancer cells [39]. Therefore, we used PC-3 and DU145 cells to investigate whether TWEAK activation of the non-canonical NF-κB pathway also occurred in these cells, or alternatively, if this effect was restricted to cell lines where TWEAK treatment inhibited invasive capacity. First, we confirmed that recombinant sTWEAK stimulated PC-3 and DU145 cell invasion under our experimental conditions. We found that this was indeed the case, with TWEAK treatment increasing PC-3 and DU145 cell invasion by ~2.1-fold and 6.5-fold, respectively (Figure 8A, 8B). Second, we treated each cell line with TWEAK for 24 hr and assayed for canonical and non-canonical NF-κB pathway activation by Western blot analysis. TWEAK had either no effect (PC-3) or increased (DU145) IκBα levels in these cells (Figure 8C). TWEAK treatment of both cell lines increased p100 to p52 processing. These results indicate that TWEAK:Fn14 engagement in PC-3 and DU145 cells preferentially activates the non-canonical NF-κB pathway.

### TWEAK-induced non-canonical NF-κB signaling in DU145 cells positively regulates cell invasion

**Figure 9:**
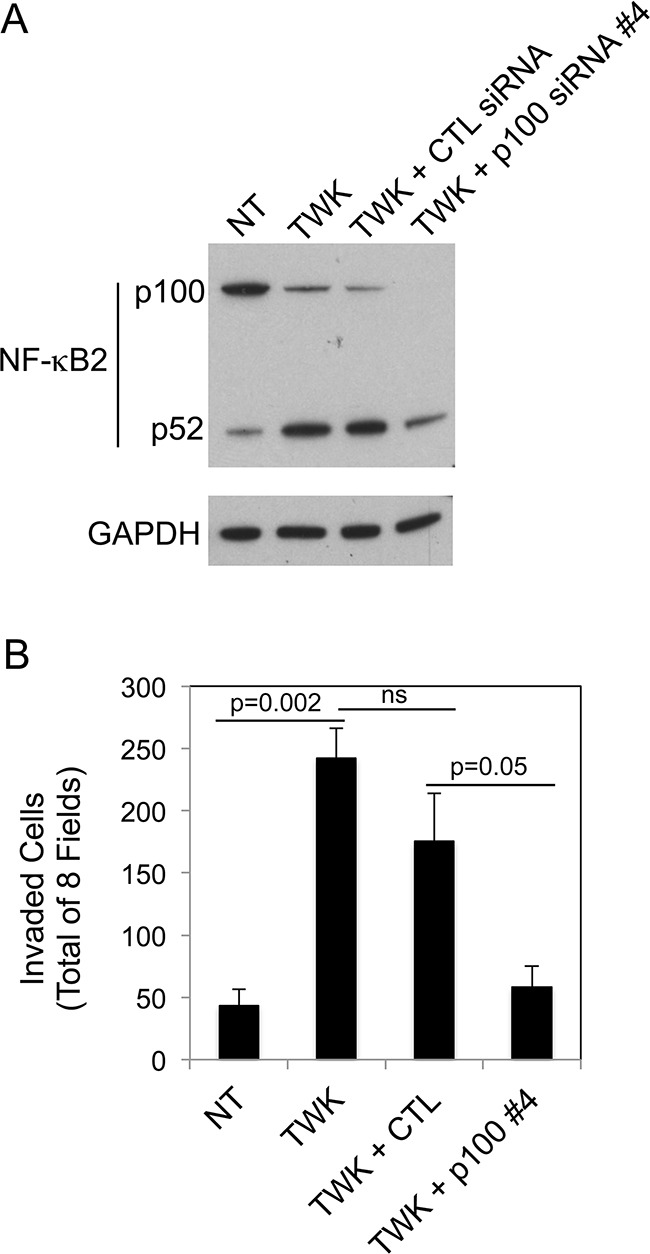
NF-κB2 p100 depletion attenuates TWEAK-stimulated DU145 cell invasion **A.** DU145 cells were either left untreated (NT, no treatment) or transiently transfected with control (CTL) siRNA or p100 siRNA #4, treated with 200 ng/ml TWEAK (TWK), and harvested 24 hr post-transfection. Lysates were prepared and NF-κB2 (p100/p52) and GAPDH levels were evaluated by Western blot analysis. This Western blot was done twice. **B.** DU145 cells were either left untreated or transiently transfected with CTL siRNA or p100 siRNA #4, treated with 200 ng/ml TWEAK, harvested 24 hr post-transfection and plated in Matrigel invasion chambers. The number of invaded cells for each invasion chamber was determined from photographs of eight randomly chosen fields. The values shown are the mean ± SEM for three chambers per condition. Significance was measured by Student's t-test (ns = not significant; p > 0.05). The invasion assay was done twice and the results of one representative experiment are shown here.

We determined if TWEAK-stimulated prostate cancer cell invasion, like TWEAK-inhibited melanoma cell invasion (Figure 6), was dependent on non-canonical NF-κB signaling. For this analysis, DU145 cells were either left untransfected, transfected with control siRNA, or transfected with our most effective p100-specific siRNA (#4). Cells were harvested and an aliquot was lysed and used to confirm effective p100 depletion by Western blot analysis (Figure 9A). The remaining cells were used for Matrigel invasion assays. We found that TWEAK treatment of control, untransfected DU145 cells increased invasion by ~5.6-fold, consistent with the data shown in Figure 8B. TWEAK treatment of the control siRNA-transfected cells resulted in an ~4.1-fold increase in invasion, a reduction of ~27% compared to the untransfected cells, but this difference was not statistically significant (Figure 9B). In contrast, TWEAK treatment of the p100-depleted cells only increased invasion by ~1.3-fold, a ~77% reduction in invasive capacity compared to the untransfected cells. This result indicates that the TWEAK-mediated increase in DU145 cell invasive capacity is due, at least in part, to non-canonical NF-κB pathway signaling.

## DISCUSSION

The TWEAK/Fn14 signaling axis has been implicated in tumor growth and metastasis and therapeutic agents that target TWEAK or Fn14 are in development for potential use in cancer patients [1, 2, 16]. However, since there is evidence that TWEAK:Fn14 binding can stimulate either “pro-tumorigenic/metastatic” or “anti-tumorigenic/metastatic” cellular responses *in vitro*, depending on the cancer cell line under investigation, it is unclear at this time whether (i) a therapeutic agent should inhibit or stimulate Fn14 signaling, and (ii) an agent can be developed for general use in all patients harboring Fn14-positive tumors, regardless of the cancer type. In this study, we provide additional evidence that TWEAK can have diverse effects on cancer cells, in this case invasive capacity, even though all the evidence to date indicates that this cytokine acts via binding to a sole signaling-competent receptor (Fn14) [60, 61]. We also show here that both TWEAK inhibition of melanoma cell invasion and TWEAK stimulation of prostate cancer cell invasion depends, at least in part, on non-canonical NF-κB pathway activation.

In a previous study, we engineered several sTWEAK-overexpressing human embryonic kidney (HEK293) cell lines and examined their growth properties *in vitro* and after subcutaneous implantation into immunodeficient mice, but no other cellular properties were examined and no signaling pathway assays were conducted [21]. Here, we engineered murine B16 melanoma cell lines that overexpressed the sTWEAK isoform and compared their cellular and molecular properties to those of matched control cell lines with low levels of endogenous TWEAK expression. The TWEAK-overexpressing cells had elevated levels of Fn14 expression, consistent with earlier reports demonstrating that TWEAK treatment of cancer cell lines increases Fn14 mRNA [48, 62] and protein [55, 62] levels and that TWEAK administration increases Fn14 gene expression in murine peritoneal tissue *in vivo* [33]. We also found that conditioned media collected from both of the TWEAK-overexpressing B16 cell lines contained higher levels of the chemokines CCL2/JE/MCP-1, CCL5/RANTES, and CXCL1/KC compared to their corresponding empty-vector B16 cell lines. This finding is consistent with prior studies using various cell types demonstrating that TWEAK:Fn14 engagement increases the expression of these three proteins [32, 44, 65–71].

Soluble TWEAK overexpression in B16 cells had an inhibitory effect on cell growth *in vitro*. A similar trend was noted in our earlier HEK293 cell study, but in this case the difference did not reach statistical significance [21]. Our finding that TWEAK has an anti-proliferative effect on melanoma cells is consistent with a prior study using the human A375 malignant melanoma cell line [3]. Although TWEAK overexpression in B16 cells inhibited cell growth *in vitro*, it had no significant effect on subcutaneous tumor growth *in vivo*. In contrast, we reported previously that TWEAK overexpression in HEK293 cells potentiated subcutaneous tumor growth and angiogenesis [21]. The main differences between these two studies that likely contribute to the different outcomes are (i) B16 cells are much more aggressive (tumorigenic) than HEK293 cells *in vivo,* and (ii) the HEK293 study was conducted using immunodeficient, *nu/nu* mice while this B16 study used syngeneic C57BL/6 mice with a fully functioning immune system.

We examined whether sTWEAK overexpression in B16 cells altered their migratory or invasive capacity. Although it has been reported that TWEAK promotes glioblastoma cell [34, 36–38] and ovarian cancer cell [35] migration in modified Boyden chamber or radial cell migration assays, we found that sTWEAK overexpression in B16 melanoma cells had no significant effect on cell motility using the scratch wound migration assay. However, both TWEAK-overexpressing B16 cells and recombinant TWEAK-treated B16 and A375 cells had greatly reduced invasive capacity in Matrigel chamber assays compared to their corresponding empty-vector cell lines or untreated cells, respectively. In the case of the four B16 cell lines, the differences in cellular invasiveness do not correlate with levels of MMP-2 and -9 activity (data not shown). Previous studies using similar Matrigel assays have shown that TWEAK treatment of cancer cells can either promote or inhibit invasion, depending on the cell line. Specifically, it has been reported that TWEAK treatment of human prostate (PC-3, DU145) [39] and ovarian (HO-8910PM) [35] cancer cells stimulates invasion, and we have confirmed the prostate cancer cell findings in this study (Figure 8). More recently, Cherry and colleagues reported that TWEAK treatment increased glioblastoma cell invasion in a 3-D collagen invasion assay [11]. In contrast to these studies, TWEAK treatment of HCT116 colorectal cancer cells resulted in a dose-dependent inhibition of invasion in a Matrigel assay [25]. Taken together, these findings demonstrate that TWEAK:Fn14 engagement can positively or negatively regulate cellular invasive activity, depending on the particular cancer cell line under investigation.

TWEAK has previously been shown to activate both the canonical and non-canonical NF-κB signaling pathways [5–11]. We found that TWEAK:Fn14 engagement in B16 cells, triggered by either high levels of sTWEAK secretion in the T1 and T2 cell lines or the addition of recombinant sTWEAK protein to V2 cells, preferentially activated the non-canonical NF-κB pathway. Furthermore, TWEAK inhibition of B16 T2 cell invasion was dependent, at least in part, on activation of this pathway. In comparison, TWEAK treatment of human A375 melanoma cells, which also inhibited invasive capacity, promoted both non-canonical and canonical NF-κB signaling. Indeed, in these cells, TWEAK treatment increased NF-κB2 (p100) expression. This finding is consistent with prior studies reporting that (i) TWEAK activation of the canonical NF-κB pathway in human mesenchymal progenitor cells induces NF-κB2 mRNA levels [31] and (ii) canonical NF-κB pathway activation positively regulates non-canonical NF-κB signaling by inducing NF-κB2 gene expression [72]. Taken together, the B16 and A375 studies demonstrate that there is not a direct correlation between TWEAK inhibition of cell invasion and preferential activation of the non-canonical NF-κB pathway.

We next examined whether TWEAK activation of the non-canonical NF-κB pathway also occurred in cells that become more invasive following TWEAK exposure; and if so, if this pathway was important for TWEAK pro-invasive activity. We used human PC-3 and DU145 prostate cancer cells to address this question since Huang and colleagues had previously reported that these cells express Fn14 and that TWEAK treatment increases their invasion in Matrigel chamber assays [39]. We found that TWEAK treatment of both cell lines preferentially activated the non-canonical NF-κB signaling pathway. Interestingly, in DU145 cells, TWEAK exposure significantly increased IκBα levels. De novo synthesis of IκBα is a well-established negative feedback loop for down-modulation of canonical NF-κB signaling [73]. Finally, we found that TWEAK stimulation of DU145 cell invasion was dependent, at least in part, on activation of the non-canonical NF-κB pathway. This finding is consistent with a recent report demonstrating that TWEAK-stimulated glioblastoma cell invasion was primarily mediated by non-canonical NF-κB pathway signaling [11].

In conclusion, we have demonstrated that TWEAK:Fn14 engagement in cancer cells can positively or negatively regulate invasive activity, depending on the particular cancer cell line under investigation. As mentioned earlier, this raises the issue of whether one wants to develop agents that block or stimulate Fn14 signaling for cancer therapy. We have also shown in this report that non-canonical NF-κB signaling pathway activation is critical for TWEAK regulation of cellular invasive capacity in B16 and DU145 cells. Although we did not detect canonical NF-κB pathway activation in these cells under our experimental conditions, we cannot exclude a role for this pathway as well. Indeed, TWEAK-stimulated ovarian cancer cell invasion can be inhibited by a pharmacological inhibitor of the canonical NF-κB pathway [35]. Finally, our finding that non-canonical NF-κB activation can have either a positive or negative downstream effect on cancer cell invasiveness *in vitro* may have potential clinical implications. Specifically, this result should be considered with regard to current efforts to develop inhibitors targeting non-canonical NF-κB pathway proteins (e.g., NIK) in order to treat cancers characterized by constitutive activation of this signaling node [14].

## MATERIALS AND METHODS

### Cell culture

The parental murine B16-BL6 melanoma cell line [57, 58] was provided by Dr. Sarah Netzel-Arnett (University of Maryland School of Medicine) and maintained in DMEM (Corning) supplemented with 10% FBS (Sigma-Aldrich), 2 mM L-glutamine and 1% penicillin-streptomycin (both from Corning). Human A375 melanoma cells (provided by Dr. David Weber, University of Maryland School of Medicine) and T98G glioma cells (American Type Culture Collection) were grown in DMEM, 10% FBS. The human PC-3 and DU145 prostate cancer cell lines were provided by Dr. Yun Qiu (University of Maryland School of Medicine) and maintained in RPMI 1640 (Corning) supplemented with 10% FBS, 2 mM L-glutamine and 1% penicillin-streptomycin. All cells were maintained at 37°C in 5% CO_2_. Cells were detached with non-enzymatic cell dissociation solution (Sigma-Aldrich) for routine passaging and experiments.

### B16 cell transfection and isolation of clonal cell lines

The pSecTag2A/Hygro expression plasmid (Invitrogen) encoding myc-tagged human soluble TWEAK was constructed and both the vector alone and this plasmid were transfected into B16 cells as described [21]. Stable B16 transfectants were selected using 400 μg/ml hygromycin B (Corning) and individual clones were screened for TWEAK expression by Western blot analysis using a myc antibody (see below). One control (vector-transfected) and one TWEAK overexpressing cell line were chosen for subsequent analysis (designated V2 and T2, respectively). The pcDNA6/His expression plasmid (Invitrogen) encoding myc-tagged human soluble TWEAK was constructed and transfected in a similar manner as above, but in this case stable vector- or TWEAK plasmid-transfected B16 cells were selected using 10 μg/ml blasticidin (Sigma-Aldrich). Following Western blot screening of individual cell clones, one control (vector-transfected) and one TWEAK overexpressing cell line was chosen for subsequent analysis (designated V1 and T1, respectively).

### Western blot analysis

For the TWEAK time-course Western blot experiment, B16-V1 cells were treated with 200 ng/ml human recombinant TWEAK (R & D Systems) for 0-24 hr in normal DMEM growth media (10% FBS) before they were harvested for Western blot analysis. Cells were harvested by scraping and lysed in HNTG lysis buffer (20 mM HEPES, 150 mM NaCl, 1.5 mM MgCl_2_, 10% glycerol, and 1% Triton X-100) supplemented with a protease inhibitor cocktail (Sigma-Aldrich) and two phosphatase inhibitor cocktails (Calbiochem). The protein concentration of each lysate was determined by BCA protein assay (Pierce Protein Biology). Equal amounts of protein were subjected to SDS-PAGE (Life Technologies) and electrotransferred to PVDF membranes (Thermo Scientific Pierce). For Western blot analysis of conditioned media samples, media was centrifuged to remove any cells and membrane fragments and then concentrated using Centricon YM-10 Centrifugal Filter Devices (Millipore). Conditioned media samples were subjected to SDS-PAGE as above. Membranes were blocked in 5% non-fat dry milk (NFDM) in TBST buffer and then sequentially incubated with the appropriate primary antibody and horseradish peroxidase (HRP)-conjugated secondary antibody (Cell Signaling Technology). The membranes were washed in TBST and then immunoreactive proteins were detected using the Amersham Enhanced Chemiluminescence Plus Kit (GE Healthcare) according to the manufacturer's instructions with CareStream film (Sigma-Aldrich). The following primary antibodies were from Cell Signaling Technology: p-ERK^T202/Y204^ (#4370), p-Akt^S473^ (#9271), NF-κB2 p100/p52 (#4882), phospho-p100^S866/870^ (#4810), phospho-p65^S536^ {#3033), phospho-IκBα^S32^ (#2859), IκBα (#9242), Fn14 (#4403), GAPDH (#2118). The anti-myc epitope antibody (9E10) was provided by Dr. Dudley Strickland (University of Maryland School of Medicine).

### TWEAK ELISA

B16 clonal cell lines were grown for 24 hr in normal DMEM growth media (10% FBS). Conditioned cell culture media was collected and stored briefly at -20°C while the cells were lysed and protein concentrations determined using the BCA protein assay. A Human TWEAK ELISA (TNFSF12) Kit (Thermo Scientific) was used to quantitate TWEAK levels in conditioned media samples (diluted 1:3; quadruplicate wells) according to manufacturer's recommendations. Absorbance readings at 410 - 550 nm (reference) were used to determine standard concentrations and plot a standard curve. Soluble human TWEAK concentrations in conditioned media were extrapolated from the curve and normalized to the protein concentration of the cell lysates.

### Secreted protein profiling using cytokine antibody arrays

The four B16 clonal cell lines were grown for 24 hr in DMEM, 0.5% FBS. Conditioned media was collected and stored overnight at -20°C. The cells were harvested and lysed, and the BCA protein assay was used to determine relative cell numbers. Appropriate amounts of conditioned media (850 - 1000 μl), normalized to protein concentrations, were then added to nitrocellulose membranes included in the Mouse XL Cytokine Antibody Array Kit (R & D Systems ARY0028). Captured proteins were detected using chemiluminescent reagents according to manufacturer's instructions and densitometry was performed to calculate relative signal intensities using Image J software.

### Cell growth assays

The B16 clonal cell lines were seeded in quadruplicate in 96-well cluster dishes (2 x 10^3^ cells for normal growth conditions and 4 x 10^3^ cells for starvation conditions) and allowed to attach. Cell media was changed to either normal DMEM growth media (10% FBS) or starvation media (0.5% FBS) and cells were allowed to grow for 72 hr before addition of WST-1 proliferation reagent (Sigma-Aldrich) according to manufacturer's recommendations. Absorbance readings at 450 - 610 nm (reference wavelength) were used to determine cellular metabolic activity.

### Subcutaneous tumor growth assays

The B16 V1, T1, V2, and T2 clonal cell lines were injected subcutaneously in the left flank (1 X 10^6^ cells per mouse) of syngeneic female, 6-8 week-old C57BL/6 mice (UMB Vet Resources; n = 7 per cell line). Subcutaneous tumor size was measured over time with digital calipers and tumor volumes were calculated with the following formula: ½ (length x width^2^).

### Scratch wound migration assays

The B16 clonal cell lines were seeded in triplicate in 6-well cluster dishes and allowed to grow to confluence in normal DMEM growth medium (10% FBS). The confluent cell monolayers were scratched in the shape of a cross using a 200 μl pipette tip and normal DMEM growth medium (10% FBS) was added to the cells. Wound closure was monitored over a 24 hr time period and photographs were taken at the four intersecting edges of the cross. Wound width at 0 and 24 hr was measured and the difference plotted as percent wound closure.

### Cell invasion assays

In the initial B16 cell line invasion experiment, the cells were untreated prior to harvesting and plating in the invasion chambers. In the subsequent experiments testing the effect of exogenously added TWEAK on V1, V2, A375, PC-3 and DU145 cell invasion, the cells were treated for 24 hr with 200 ng/ml recombinant human TWEAK (R & D Systems) before they were harvested and plated in the invasion chambers. For the B16-T2 cell NF-κB2 p100 depletion experiment, cells were transfected with siRNA (see below) for 24 hr before they were harvested and plated in the invasion chambers. For the DU145 cell NF-κB2 p100 depletion experiment with TWEAK treatment, the cells were transfected with siRNA, treated 1 hr later with 200 ng/ml TWEAK, harvested 24 hr post-transfection, and plated in the invasion chambers.

All cell treatments were performed in normal DMEM growth media (10% FBS) before the cells were harvested with non-enzymatic cell dissociation solution, re-suspended in media containing 0.5% serum (with or without 200 ng/ml TWEAK) and seeded in triplicate or quadruplicate in cell culture chambers containing 8 um-pore sized PET membranes pre-coated with growth factor-reduced Matrigel (Corning). Each cell line was seeded onto Matrigel chambers at different densities, with V1 and T1 at 2 x 10^5^, V2 and T2 at 1 x 10^5^, A375 at 1 x 10^5^, PC-3 at 1 x 10^5^, and DU145 at 1 x 10^4^ cells per chamber. The chambers were then placed in 24-well plates (Corning) with growth media containing 10% FBS (B16 and A375) or 20% FBS (PC-3 and DU145) as a chemoattractant. Cells were allowed to invade for 20 hr and then fixed with 20% methanol and stained with 0.5% crystal violet. Pictures were taken of eight randomly chosen fields at 20X magnification under a light microscope for each membrane. Cells were counted using ImageJ software and summed to calculate total number of cells invaded.

### Small interfering RNA transfections

Cells were plated, allowed to attach for 5 hr, and then transfected using Lipofectamine RNAiMax transfection reagent (Invitrogen) according to the manufacturers’ instructions with either negative control siRNA, Fn14 siRNA #1 or #4 targeted to the mouse Fn14 transcript (all three siRNAs at a final concentration of 20 nM), or negative control siRNA, p100 siRNA #3 or #4 targeted to the mouse NF-κB2 (p100) transcript (all three siRNAs at a final concentration of 30 nM). All siRNAs were purchased from Qiagen: negative control (#1022076), mouse Fn14 (#SI01452311, #SI01452332), mouse p100 (#SI01327025, #SI01327032). It was not necessary to purchase human p100 siRNAs for the human cancer cell experiments because we found that the mouse p100 siRNAs could also deplete human p100 protein. Cells were harvested at 24 hr post-transfection and seeded onto Matrigel invasion chambers as described above, with the remaining cells harvested at either 24 or 48 hr post-transfection for Western blot analysis.

### Statistical analysis

Results are all presented as mean ± SEM and the two-sample Student's t-test was used to determine statistical significance. P-values <0.05 were considered significant.

## SUPPLEMENTARY MATERIALS FIGURES


