# Supplementary material for: TWEAK activation of the non-canonical NF-κB signaling pathway differentially regulates melanoma and prostate cancer cell invasion
Source: Oncotarget. 2016 Nov 3;7(49):81474–92. doi: 10.18632/oncotarget.13034 (PMC5348407; doi:10.18632/oncotarget.13034)
Supplement: Supplementary file 1 [file oncotarget-07-81474-s001.pdf]

TWEAK activation of the non-canonical NF-κB signaling pathway differentially regulates melanoma and prostate cancer cell invasion

SUPPLEMENTARY FIGURES

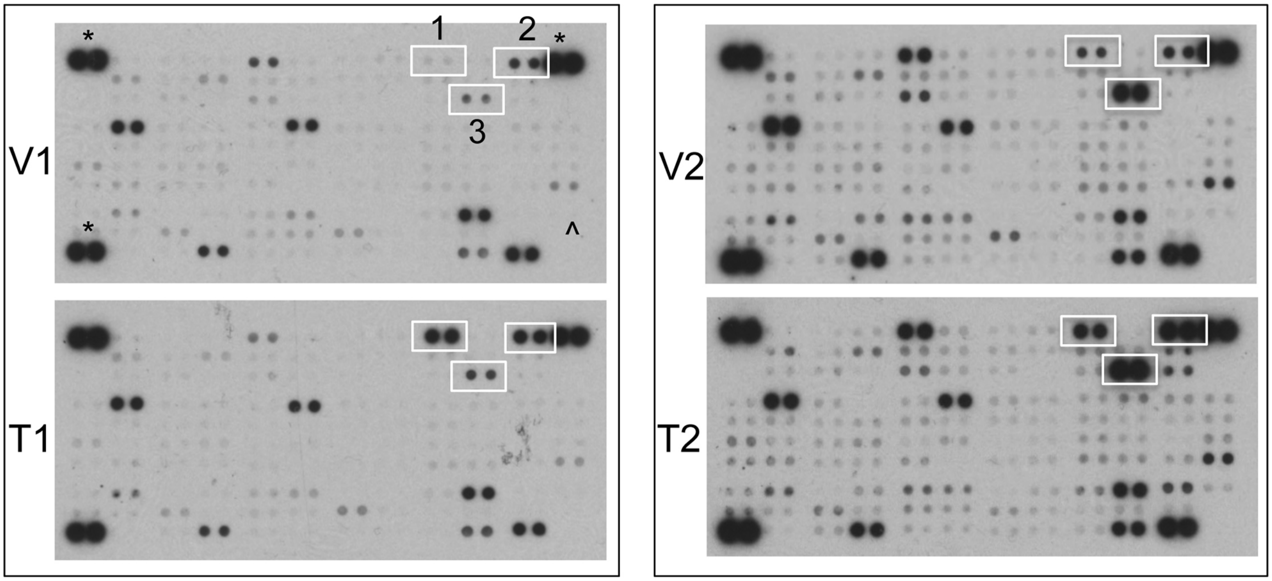

Supplementary Figure S1: Effect of TWEAK overexpression in B16 cells on cytokine/ chemokine expression. Normalized amounts of B16 V1, V2, T1, and T2 cell conditioned media sample were added to Mouse XL Cytokine Antibody membranes containing 111 capture antibodies spotted in duplicate (R & D Systems). Immunoreactive proteins were detected according to manufacturer's instructions. On membrane V1, positive and negative control reference spots are indicated with an asterisk and a caret sign, respectively. Spots corresponding to three TWEAK-inducible chemokines are boxed on all four membranes and identified by numbers on V1. 1= CCL2/JE/MCP-1, 2= CCL5/RANTES, 3= CXCL1/KC. Increased expression of these three proteins was also detected using cell lysates and another cytokine antibody array containing fewer capture antibodies (Mouse Cytokine Array Kit, Panel A (R & D Systems)).

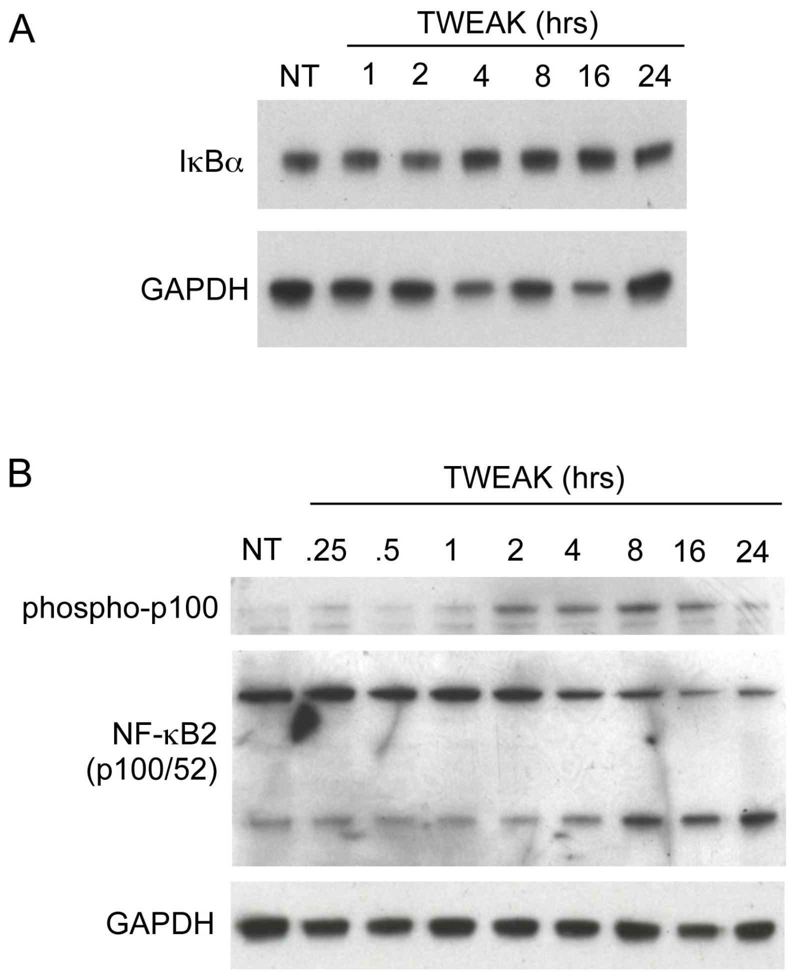

Supplementary Figure S2: Effect of TWEAK treatment on B16 V1 melanoma cells. Cells were either left untreated (NT, no treatment) or treated with 200 ng/ml TWEAK for the indicated time periods. Cells were harvested and IκBα and GAPDH levels **(panel A)** or phospho-p100, NF-κB2 (p100/52), and GAPDH levels **(panel B)** were analyzed by Western blotting. These Western blots were done two times.
